# Preclinical PET imaging of EGFR levels: pairing a targeting with a non-targeting Sel-tagged Affibody-based tracer to estimate the specific uptake

**DOI:** 10.1186/s13550-016-0213-8

**Published:** 2016-07-07

**Authors:** Qing Cheng, Helena Wållberg, Jonas Grafström, Li Lu, Jan-Olov Thorell, Maria Hägg Olofsson, Stig Linder, Katarina Johansson, Tetyana Tegnebratt, Elias S. J. Arnér, Sharon Stone-Elander, Hanna-Stina Martinsson Ahlzén, Stefan Ståhl, Sel-tag imaging project

**Affiliations:** Department of Medical Biochemistry and Biophysics, Karolinska Institutet, Stockholm, Sweden; Department of Clinical Neurosciences, Karolinska Institutet, Stockholm, Sweden; Karolinska Experimental Research and Imaging Center, Department of Comparative Medicine, Karolinska University Hospital, Stockholm, Sweden; Neuroradiology Department, R3:00, Karolinska University Hospital, SE-17176 Stockholm, Sweden; Department of Oncology and Pathology, Karolinska Institutet, Stockholm, Sweden; Division of Protein Technology, School of Biotechnology, Royal Institute of Technology, Stockholm, Sweden

**Keywords:** Epidermal growth factor receptor, Affibody molecule, Sel-tag, Dual tracer imaging, Positron emission tomography

## Abstract

**Background:**

Though overexpression of epidermal growth factor receptor (EGFR) in several forms of cancer is considered to be an important prognostic biomarker related to poor prognosis, clear correlations between biomarker assays and patient management have been difficult to establish. Here, we utilize a targeting directly followed by a non-targeting tracer-based positron emission tomography (PET) method to examine some of the aspects of determining specific EGFR binding in tumors.

**Methods:**

The EGFR-binding Affibody molecule Z_EGFR:2377_ and its size-matched non-binding control Z_Taq:3638_ were recombinantly fused with a C-terminal selenocysteine-containing Sel-tag (Z_EGFR:2377_-ST and Z_Taq:3638_-ST). The proteins were site-specifically labeled with DyLight488 for flow cytometry and ex vivo tissue analyses or with ^11^C for in vivo PET studies. Kinetic scans with the ^11^C-labeled proteins were performed in healthy mice and in mice bearing xenografts from human FaDu (squamous cell carcinoma) and A431 (epidermoid carcinoma) cell lines. Changes in tracer uptake in A431 xenografts over time were also monitored, followed by ex vivo proximity ligation assays (PLA) of EGFR expressions.

**Results:**

Flow cytometry and ex vivo tissue analyses confirmed EGFR targeting by Z_EGFR:2377_-ST-DyLight488. [Methyl-^11^C]-labeled Z_EGFR:2377_-ST-CH_3_ and Z_Taq:3638_-ST-CH_3_ showed similar distributions in vivo, except for notably higher concentrations of the former in particularly the liver and the blood. [Methyl-^11^C]-Z_EGFR:2377_-ST-CH_3_ successfully visualized FaDu and A431 xenografts with moderate and high EGFR expression levels, respectively. However, in FaDu tumors, the non-specific uptake was large and sometimes equally large, illustrating the importance of proper controls. In the A431 group observed longitudinally, non-specific uptake remained at same level over the observation period. Specific uptake increased with tumor size, but changes varied widely over time in individual tumors. Total (membranous and cytoplasmic) EGFR in excised sections increased with tumor growth. There was no positive correlation between total EGFR and specific tracer uptake, which, since Z_EGFR:2377_ binds extracellularly and is slowly internalized, indicates a discordance between available membranous and total EGFR expression levels.

**Conclusions:**

Same-day in vivo dual tracer imaging enabled by the Sel-tag technology and ^11^C-labeling provides a method to non-invasively monitor membrane-localized EGFR as well as factors affecting non-specific uptake of the PET ligand.

**Electronic supplementary material:**

The online version of this article (doi:10.1186/s13550-016-0213-8) contains supplementary material, which is available to authorized users.

## Background

The overexpression of epidermal growth factor receptor (EGFR) in many human tumors [1] has been related to metastasis, therapy resistance, and poor prognosis [2]. Extracellular, intracellular, and mutated EGFRs have been therapeutic targets in cancer for some time [3] and the ability to quantify expression levels could be used to identify patients that would benefit from anti-EGFR therapies. However, poor correlations between conventional assessments of EGFR expression and clinical responses have raised questions about whether it is the assays that are inadequate or whether there are fundamental issues related to the in vivo function of EGFRs that need to be considered [4]. Non-invasive nuclear medicine-based molecular imaging of receptors can potentially offer advantages over conventional biopsy-based analyses since these methods provide global tumor assessments that should be less prone to sampling errors. Radiolabeled tracers ranging from small molecules up to antibodies have been developed for EGFR imaging using single photon and positron emission tomography (SPECT and positron emission tomography (PET), respectively) [5]. However, there is still no consensus on the most appropriate radiotracer to use. Most of the tracers studied have had issues concerning in vivo specificity, selectivity, and/or sensitivity. In addition to variations in non-targeting uptakes due to the varying characteristics of individual tumors in which the receptor modeling is to be performed, the imaging results have also been affected by metabolism, limited bioavailability, and inappropriate kinetics of the imaging probes [6].

Dual or paired tracer imaging has been used in neuroreceptor imaging since the mid-1980s to assess non-targeting contributions to the signal of targeting radiotracers, but to a much less extent for receptor imaging in tumors. Most of the studies pairing tracers in the same tumor have either been performed in SPECT studies in the brain or in preclinical optical imaging of surface tumors [7]. We aimed here to examine some of the issues reported in EGFR imaging by using a dual tracer imaging strategy based on Affibody molecules and preclinical PET. Affibody molecules are small size proteins (58 residues, ≈7 kDa) obtained by randomizing thirteen surface residues in the Affibody protein scaffold [8]. High-affinity binders have been developed and evaluated for SPECT or PET imaging of tumors [9]. Several probes targeting EGFR have been developed [10, 11]. In particular, Z_EGFR:1907_ has been labeled for preclinical SPECT (^111^In) and PET (^64^Cu, ^18^F) applications [12–14]. Subsequently, Z_EGFR:2377_, which binds with equally high (subnanomolar) affinity to human and murine EGFR, was developed so that the tracer behavior in rodents would be more analogous to that expected in humans [15]. Z_EGFR:2377_ was labeled with ^111^In for SPECT studies, and EGFR-expressing tumors were successfully imaged when the tracer specific activity was appropriately adjusted to reduce competing uptake in non-tumor tissues [16]. Fluorescently labeled targeting and non-targeting Affibody molecules have also recently been co-injected in preclinical fluorescence imaging studies in order to estimate and correct for non-specific contributions to the EGFR expression levels determined with the targeting Affibody molecule [17].

Here, we used small animal PET and short-lived, dual targeting and non-targeting Affibody molecules to examine some of the parameters affecting the in vivo quantification of EGFR receptors. We paired Z_EGFR:2377_ with a size-matched control Z_Taq:3638_ (S1-1 in [18]) to perform in vivo studies of binding to EGFR-expressing xenografts. To examine the ability of Z_EGFR:2377_ to detect different EGFR expression levels in tumors with varying characteristics, we used two models (A431 (epidermoid carcinoma) and FaDu (squamous cell carcinoma)) and, in one series, examined changes in tracer uptake with the growth of A431 tumors. Labeling was performed with ^11^C (*t*_1/2_ = 20.4 min). This permitted observation in vivo for 60 min, which is consistent both with the fairly rapid clearance of Z_EGFR:2377_ [16] and the desirability for performing “same-day” sequential imaging with the control Z_Taq:3638_ in each individual. A Sel-tag (ST) containing a selenocysteine residue within a C-terminal -Gly-Cys-Sec-Gly sequence [19] was used for the rapid and site-specific labeling with ^11^C, giving the radiotracers [methyl-^11^C]-Z_EGFR:2377_-ST-CH_3_ and [methyl-^11^C]- Z_Taq:3638_-ST-CH_3_. Our results demonstrate the feasibility and potential advantages of same-day in vivo dual tracer imaging of membrane EGFR levels using ^11^C-labeled Affibody molecules and suggest this PET approach to be useful for non-invasively evaluating specific and unspecific retention of labeled probes in peripheral tumors with varying target expressions.

## Results

### Preparation and labeling of the Sel-tagged Affibody molecules

The Sel-tagged Affibody molecules were successfully obtained by expression in *E. coli* as C-terminal fusions to green fluorescent protein (GFP), then recovered with immobilized metal ion affinity chromatography (IMAC), released by tobacco etch virus (TEV)-protease cleavage, and purified by high-performance liquid chromatography (HPLC). Correct expected masses (7.267 and 7.157 kDa for Z_EGFR:2377_-ST and Z_Taq:3638_-ST, respectively) were verified by electrospray ionization-mass spectroscopy.

Using the previously developed protocol [20], Affibody molecules were ^11^C-labeled and purified within 50 min, with decay-corrected yields up to 20 % based on used [^11^C]methyl iodide (CH_3_I). Radiochemical purities were 95 ± 3 %, with labeled dimer occasionally detected. Efforts were not made to increase the specific radioactivity since an optimal rather than the highest possible specific activity was required [13, 14, 16].

### EGFR targeting by Z_EGFR:2377_ -ST but not Z_Taq:3638_-ST

**Fig. 1:**
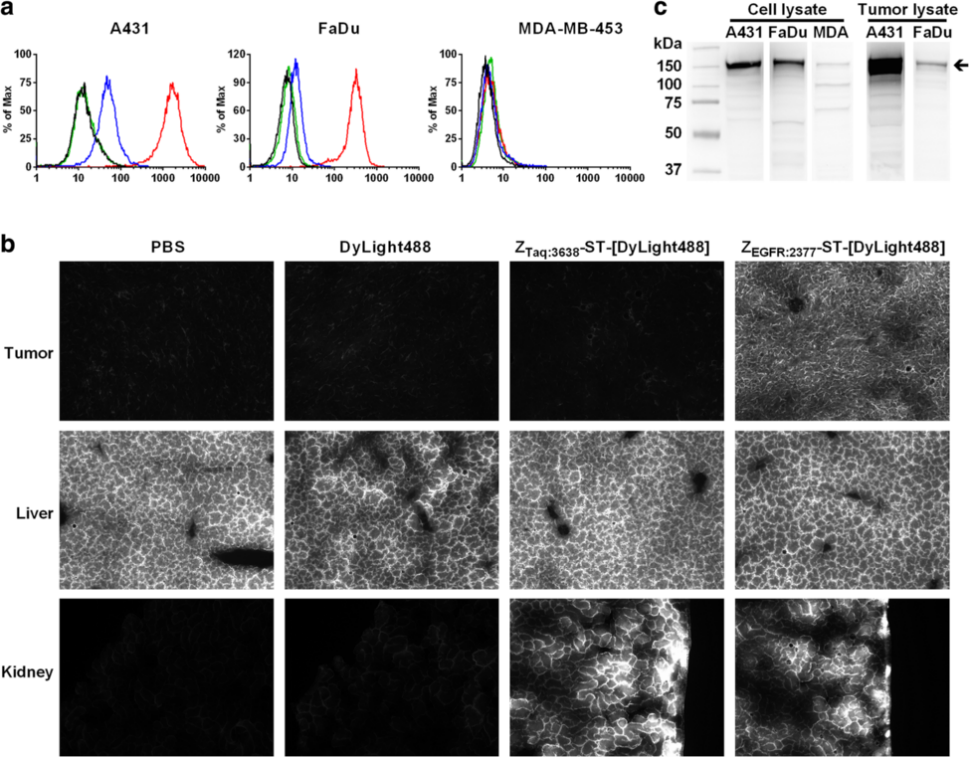
**a** Cell-binding assay of non-blocked (*red*) and blocked (*blue*, tenfold Z_EGFR:2377_) Z_EGFR:2377_-ST-[DyLight 488] and controls Z_Taq:3638_-ST-[DyLight 488] (*green*) and DyLight 488 dye (*black*) using FACS analysis in A431 (*left*), FaDu (*middle*), and MDA-MB-453 (*right*) cells with high, medium, and low/no expressions of EGFR, respectively. **b** Fluorescent microscopy images of tumor (A431), liver, and kidney. Fluorescence in the tumor is only observed with the Z_EGFR:2377_ probe. High autofluorescence of the liver is observed with all probes. Fluorescence from both the Z_EGFR:2377_ and Z_Taq:3638_ probes is observed in the kidney. **c** western blots of A431, FaDu, and MDA from cell (*left*) and tumor (*right*) lysates using an antibody against human EGFR. The protein concentration of the cell lysates was determined by Bradford protein assay and Ponceau S staining of the membranes was used as loading control. The *arrow* indicates full-length EGFR

In vitro and ex vivo assays were used to test whether the C-terminal ST and labeling at the ST interfered with the EGFR binding of Z_EGFR:2377_ (characterized in [16]). Flow cytometry (Fig. 1a) showed Z_EGFR:2377_-ST-[DyLight488] (red curves) clearly bound to A431 and less to FaDu, but not at all to MDA-MB-453 cells (human breast carcinoma), which correlated well with EGFR levels (western blot, Fig. 1c). Z_EGFR:2377_-ST-[DyLight488] binding in A431 and FaDu cells was significantly reduced by blocking with excess Z_EGFR:2377_ (blue curves). Non-targeting Z_Taq:3638_-ST-[DyLight488] (green curves) showed no binding.

In vivo targeting ability was compared by analyzing sections of tissues excised from mice that had been injected with the fluorescently labeled Affibody molecules. Fluorescence in the tumors was only detected with Z_EGFR:2377_-ST-[DyLight488], indicating the probe’s capability for EGFR targeting in vivo (Fig. 1b, top panels). Binding in the liver (Fig. 1b, middle panels) could not be evaluated due to autofluorescence [21]. Fluorescence from both tracers was observed in the kidneys (Fig. 1b, lower panels), in agreement with renal elimination of Affibody molecules [9].

### In vivo biodistribution of [methyl-^11^C]-labeled Z_EGFR:2377_ –ST-CH_3_ and Z_Taq:3638_-ST-CH_3_

**Fig. 2:**
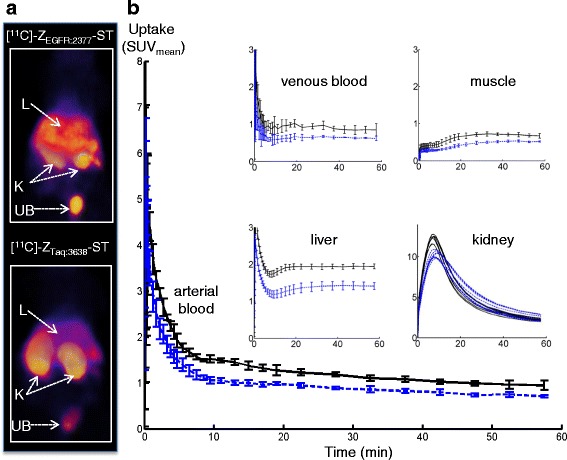
Biodistribution of [methyl-^11^C]-Z_EGFR:2377_-ST-CH_3_ and [methyl-^11^C]-Z_Taq:3638_-ST-CH_3_ in healthy Balb/c mice (*black lines* Z_EGFR:2377_ and *blue lines* Z_Taq:3638_ (means ± SD, *n* = 4 mice): **a** PET images (supine, summed from 30–60 min, 3-D volume rendering) and **b** time activity curves (SUV) in arterial and venous blood, skeletal muscle, liver, and kidney over 60 min after injection

Key features of the tracers’ distributions in healthy mice during 60 min are shown in Fig. 2. The amount of protein injected was adjusted by spiking with unlabeled protein. Two protein amounts, 27 and 49 μg, were used in accordance with previous studies [14, 16], to demonstrate that the large uptake in the liver could be at least partially blocked so tracer would be available for tumor targeting. EGFR blocking was tolerated well with the Affibody molecules, since they are antagonistic or pharmacologically neutral [16]. Even after the partial blocking, larger uptakes of [methyl-^11^C]-Z_EGFR:2377_-ST-CH_3_ were still observed in the liver (Fig. 2a). The time-activity curves (TACs) were very similar for the two masses injected, and the standard deviations were similar to those of the non-spiked [methyl-^11^C]-Z_Taq:3638_-ST-CH_3_ (Fig. 2b). Therefore, instead of the four individual curves, one curve of the means of the four animals with the standard deviations is shown here. Radioactivity in the arterial and venous blood began to plateau after 40 min. Levels for [methyl-^11^C]-Z_EGFR:2377_-ST-CH_3_ were higher than for [methyl-^11^C]-Z_Taq:3638_-ST-CH_3_. This observation is consistent with the binding of the targeting ligand to shed or soluble EGFR, to smooth muscle cells in the blood vessels and/or tracer dissociating from EGFR receptors and returning to the circulation [16]. The higher uptake for the targeting ligand in the muscle may be due to differences in blood radioactivity since there are no or very low EGFR levels in skeletal muscle and bone marrow.

Liver radioactivity plateaued after 30 min but was 30 % higher with [methyl-^11^C]-Z_EGFR:2377_-ST-CH_3_, since the hepatic EGFR binding is not completely blocked at these doses [14, 16]. Contributions from blood pool, non-specific uptake, and/or tracer metabolism are revealed by the [methyl-^11^C]-Z_Taq:3638_-ST-CH_3_ uptake. The highest radioactivity concentrations were found in the kidneys and urinary bladder. The higher and slightly earlier peak and a tendency to a faster renal washout observed for [methyl-^11^C]-Z_EGFR:2377_-ST-CH_3_ may be due to the added protein and/or the slightly more positive charge of the control [22]. Low uptakes of radioactivity were measured ex vivo in the lung, pancreas, spleen, stomach, and intestines, but their relative amounts could not be analyzed quantitatively in vivo in the mice due to the influence of partial volume effects (PVEs).

### In vivo tumor studies

**Fig. 3:**
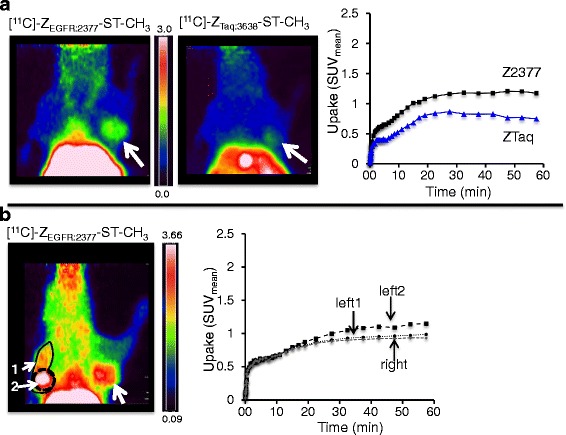
PET images, summed 30–60 min, and TACs from a SCID mouse (prone) bearing tumors (*white arrows*): **a** one FaDu xenograft (1 × 10^6^ cells, 12 days) or **b** two FaDu xenografts (*left*: (1 × 10^6^ cells, 12 days); *right*: (0.5 × 10^6^ cells, 12 days). Comparison A illustrates the higher uptake with targeting [methyl-^11^C]-Z_EGFR:2377_-ST-CH_3_ but with a ≈60 % non-targeting uptake of [methyl-^11^C]-Z_Taq:3638_-ST-CH_3_. Comparison B illustrates the visually discernable heterogeneous uptake of the targeting Affibody in the larger tumor on the left. SUV_mean_ is affected by whether the entire (1) or only central ROI (2) of the left tumor is used

**Fig. 4:**
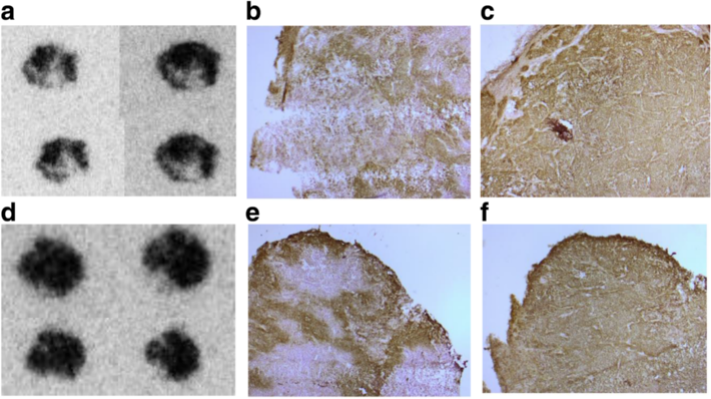
Ex vivo analyses of sections of the two tumors in Fig. 3b (*left tumor* (**a**–**c**); *right tumor* (**d**–**f)**). **a**, **d** Phosphoimaging of sections of tumors excised immediately after the 60 min PET scan with [methyl-^11^C]-Z_EGFR:2377_-ST-CH_3_. **b**, **e** IHC detecting CAIX, staining was performed on formalin-fixed, paraffin-embedded material using antibody against CAIX (BioScience, Slovakia) at a dilution 1:500. **c**, **f** IHC detecting EGFR, staining was performed on formalin-fixed, paraffin-embedded material using antibody against EGFR (Sigma, Sweden) at a dilution 1:800

Uptakes of [methyl-^11^C]-Z_EGFR:2377_-ST-CH_3_ in FaDu xenografts were generally low (standardized uptake values (SUV)_mean_ = 0.64–1.22) and plateaued after 30 min (Fig. 3a). Uptakes of the control (SUV_mean_ = 0.68–0.79) varied from one half to nearly the same levels as that of the targeting protein. Uptake of [methyl-^11^C]-Z_EGFR:2377_-ST-CH_3_ was heterogeneous in larger FaDu tumors (Fig. 3b, left side). Even when areas with lower uptake were excluded by thresholding, the SUV_mean_ for the left and right tumors in this animal only differed by ≈15 %. Similar results were obtained for one of the other three animals while the larger tumors in the two remaining mice were too necrotic for reasonable comparisons of uptakes between the xenografts. Phosphoimaging (PI) of sections of excised tumors from Fig. 3b confirmed the heterogeneous distribution of the targeting tracer seen in the PET images, in spite of immunohistochemistry (IHC) staining results that indicated fairly homogenous EGFR expressions (Fig. 4a, c vs. d, f). High levels of carbonic anhydrase IX (CAIX) immunostaining (Fig. 4b vs. e) indicated the presence of large hypoxic areas.

**Fig. 5:**
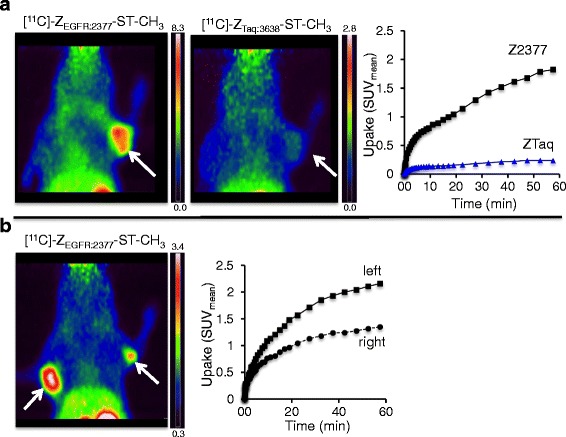
PET images, summed 30–60 min, and TACs from a Balbc nu/nu mouse (prone) bearing tumors (*white arrows*): **a** one A431 xenograft (1 × 10^7^ cells, 15 days) or **b** two A431 xenografts (*left*: 1 × 10^7^ cells, 28 days; *right*: 1 × 10^7^ cells, 25 days). Comparison A shows a 7-times higher uptake with targeting [methyl-^11^C]-Z_EGFR:2377_-ST-CH_3_ compared to the non-targeting [methyl-^11^C]-Z_Taq:3638_-ST-CH_3_. Comparison B illustrates uptake of the targeting Affibody increasing as the tumors grow from time from inoculation

In A431 xenografts, uptakes of [methyl-^11^C]-Z_EGFR:2377_-ST-CH_3_ were higher than in FaDu (Fig. 4) (SUV_mean_ = 0.78–2.49) while uptakes of the control were usually lower (SUV_mean_ = 0.22–0.86; one particular outlier with SUV_mean_ = 1.28). In one example (Fig. 5a), the uptake of [methyl-^11^C]-Z_EGFR:2377_-ST-CH_3_ was ≈7 times higher than that of [methyl-^11^C]-Z_Taq:3638_-ST-CH_3_. The uptake of [methyl-^11^C]-Z_EGFR:2377_-ST-CH_3_ generally increased with the tumor size (Fig. 5b) and appeared to be homogenous in the size range studied here. Interestingly, the SUVs were still increasing at 60 min, indicating that equilibrium was not yet attained, while that of [methyl-^11^C]-Z_Taq:3638_-ST-CH_3_ leveled off from 35 min. Performing acquisitions over more than 60 min to probe whether the increases eventually leveled off was not possible due to the low counting statistics after three times the half-life of the radionuclide.

**Fig. 6:**
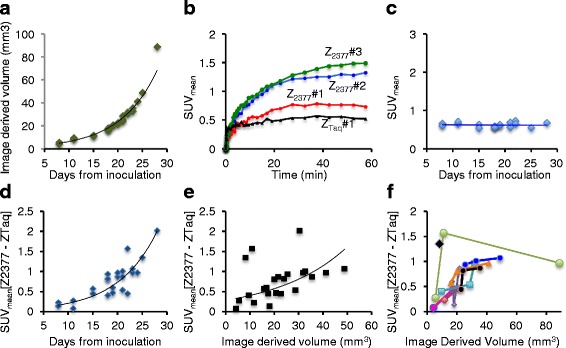
Data from the longitudinal study of A431 xenografts: **a** Changes in tumor volumes with the days since inoculation. **b** TACs of the differing uptakes in one A431 xenograft of [methyl-^11^C]-Z_Taq:3638_-ST-CH_3_ (*black* curve, day 8) and targeting [methyl-^11^C]-Z_EGFR:2377_-ST-CH_3_ (*red*, *blue*, and *green curves* on days 8, 19, and 21, respectively). **c** SUV_mean_ for the uptake of [methyl-^11^C]-Z_Taq:3638_-ST-CH_3_ measured in the A431 tumors vs. days since inoculation. **d** Variations in specific uptake (SUV_mean(ZEGFR:2377)_–SUV_mean(ZTaq:3638)_) with the growth of all ten xenografts from inoculation. **e** Variations in specific uptake (SUV_mean_ of Z_EGFR:2377_–SUV_mean_ of Z_Taq:3638_) of all ten xenografts as their volumes increased. **f** Variations in specific uptake, SUV_mean_, in individual xenografts as their volumes increased

In the longitudinal study of ten tumors in five mice (growths as in Fig. 6a), the uptake of [methyl-^11^C]-Z_EGFR:2377_-ST-CH_3_ equilibrated quickly when the tumors were small but was still increasing at 60 min when the tumors had grown larger (Fig. 6b, blue and green vs. red curves), which suggests that the time of observation needed until maximum uptake can vary with tumor development. The uptake of [methyl-^11^C]-Z_Taq:3638_-ST-CH_3_ (0.6 ± 0.1) plateaued and did not, in this series, notably change with tumor size (Fig. 6c). Grouping all scans for the five mice and ten tumors together, the increasing specific uptake (i.e., [SUV_ZEGFR:2377_–SUV_ZTaq:3638_]) correlated (exponential regression) with the number of days from the time from inoculation for both SUV_mean_ (Fig. 6d, *r*^2^ = 0.73) and SUV_max_ (*r*^2^ = 0.70, data not shown). The variance was much greater in the correlations to the xenograft volumes (SUV_mean_ (Fig. 6e, exponential regression, *r*^2^ = 0.27 (the largest tumor was here treated as an outlier and excluded from the plot)) and SUV_max_ (*r*^2^ = 0.38, data not shown)), indicating the correlation to be quite poor. However, several PET studies were performed longitudinally in this study, which took advantage of the strength of in vivo imaging for observing changes on the level of each individual tumor instead of analysing by group that is typical of most ex vivo studies. It was obvious from the monitoring over time that changes in the SUVs were somewhat different for each xenograft. There was a sharp increase in SUVs between the first and second imaging in the very small xenografts, but, as they grew larger, the incremental SUV changes between the second and third experiments were smaller or, in some cases, even decreased (Fig. 6f).

**Fig. 7:**
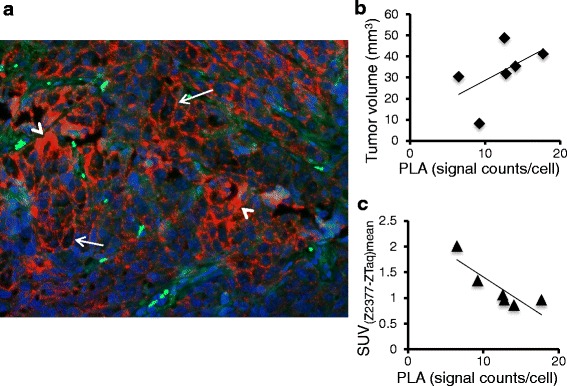
In situ PLA analyses of EGFR in formalin-fixed, paraffin-embedded sections of A431 xenografts from the longitudinal study. **a** EGFR-positive tissue, magnification ×20. *White arrows* and *arrowheads* indicate membranous and cytoplasmic patterns of EGFR immunostaining, respectively. *Red dots*: EGFR single proteins, *blue*: Hoechst nuclear staining, and *green*: auto fluorescence. **b** PLA signal counts/cell (relative EGFR expression level) in EGFR positive sections vs. volumes of the excised tumors. **c** Specific tracer uptake on the last day of imaging vs. the PLA signal counts/cell in excised tumor sections

Proximity ligation assay (PLA) analyses of all the sections of six of the harvested tumors showed positive and negative areas with membranous (Fig. 7a, arrows) as well as cytoplasmic (Fig. 7a arrowheads) EGFRs detected. The PLA signals in the tumor sections were generally greater in tumors of larger volumes, but there was quite a large variance and therefore the correlation is considered poor (Fig. 7b, linear regression, *r*^2^ = 0.29). There was, however rather clearly, a negative relation between the PLA signals and the SUVs measured on the final PET imaging day (Fig. 7c, linear regression, *r*^2^ = 0.74).

## Discussion

This study is, to our knowledge, the first longitudinal in vivo study in tumor xenografts using a radiolabeled EGFR-binding tracer combined with an analogously labeled size-matched control. The short-lived ^11^C radionuclide enabled same day, paired imaging in each individual tumor. Therefore, the protocol could minimize influences on the results due to changing tumor characteristics over time (compared to when studies are instead performed on same xenograft on different days) or from differing characteristics of different xenografts (when the comparisons are instead performed in different animals). The Sel-tag enabled labeling at the same site with fluorescent and radioactive labels, which allowed evaluations of the tracers’ EGFR binding capabilities in vitro, ex vivo, and in vivo. Labeling was fast, and yields obtained even with ^11^C were sufficient for the intended use. Z_EGFR:2377_ had an appropriate affinity and size to quickly distribute from the circulation to the tumor. The results obtained using this dual tracer protocol revealed that the non-specific uptake should be evaluated, especially when receptor levels are low and influences on tracer behavior from other tumor characteristics are comparatively large, and that EGFR levels targeted by a membrane-binding radioligand do not directly correlate to the total (extra- plus intracellular) levels.

Z_EGFR:2377_ and Z_Taq:3638_ were expected to distribute similarly since they are essentially the same size, charges at physiological pH are nearly neutral and differ only by two (two more lysines in Z_Taq:3638_), and they have an equal number of hydrophobic acids and essentially the same hydropathicity (Affibody AB, personal communication). Differences were, however, observed in their concentrations in blood and several organs in vivo, which were interpreted to be primarily related to Z_EGFR:2377_ binding to peripheral EGFR. If receptor binding is to be quantified with PET, an input function based on either radioactivity in arterial blood or a reference region devoid of receptors is needed [23]. For such studies, it will be important to determine the identity of the radioactivity in the blood. At least part of the Z_EGFR:2377_-related radioactivity in blood delivered to the tumors after 50–60 min apparently has EGFR-binding capability, since its uptake, but not that of the control, continued to increase in the larger A431 tumors (Figs. 5 and 6b, c). Blood sampling with subsequent analyses coupled to image-derived blood curves could determine how much of (and when) the radioactivity is intact, free, or bound tracer. These studies are, however, preferably performed in larger animals since the very small blood volumes in mice limit both the sampling frequency and volumes that can be taken for the analyses.

Affibody molecules are ≈7 kDa in size. Even though enhanced permeability and retention (EPR) properties of tumors are primarily utilized to enhance delivery of drugs >40 kDa, smaller proteins are also more extravasated in highly vascularized tumors and retained longer due to their impaired lymphatics [24]. The control protein, [methyl-^11^C]-Z_Taq:3638_-ST-CH_3_, distributed immediately (the vascular input) to both tumors studied here, but additional increases in uptake over ≈30 min (see e.g. Figs. 3a and 5b) suggest a passive EPR-related retention also of these small proteins during the 1-hr observation period. The non-specific uptake could sometimes be relatively high; it varied between tumor models and was not always the same for xenografts of the same cell line. Both FaDu and A431 xenografts are highly angiogenic [25, 26], and new blood vessels therefore grow rapidly and eventually become disorganized, dilated, and leaky. There are considerable inter- and intratumoral heterogeneities in the vascular volumes of tumors of different angiogenic states [27]. Contributions to tracer behavior from non-specific uptake due to the vascular properties in individual tumors need to be accounted for since they may be quite large and may change over time and during therapy [28]. This was simplest done here (in Fig. 6d–f) by subtracting the TACs of the non-targeting ligand from that of the targeting to give a measure of the specific uptake. Compartmental modeling can be used to further quantify the binding and non-specific uptakes (e.g., [29, 30] reviewed in [7]). However, TACs from several of these studies (e.g., Figs. 5 and 6b) indicate that the uptake of the targeting tracer did not always reach equilibrium during the 1-hr scan (the limit for the observation time due to the short half-life of ^11^C). Mathematical models can be used to quantify irreversibly binding tracers, but they would require an input from arterial plasma [31], which, as discussed above, is far more readily sampled in animals larger than mice. Kinetic analyses of the uptakes of the probe labeled with a longer-lived radionuclide might provide additional information about if or when binding equilibrium is achieved. However, in this case, a same-day dual tracer approach to also estimate the non-specific uptake could probably only be done if, for example, the ligands were labeled with different radionuclides and monitored with SPECT.

Other tumor characteristics will also affect quantifications of tracer uptakes. This is illustrated with the FaDu xenografts imaged here. Here, uptakes of the targeting tracer were low (consistent with low expression levels) but were also visibly intra- and inter-tumorally heterogeneous. The uptake heterogeneity was inconsistent with the fairly homogenous EGFR expressions shown by the ex vivo IHC but could be consistent with the prominent hypoxia levels detected (Fig. 4). Many experimental tumors grow rapidly and quickly become hypoxic. Vessels in hypoxic regions collapse due to increasing pressure in growing tumors. A probe’s targeting ability in vivo will thus be affected, since the number of functional vessels delivering the probe has decreased and the distance the probe must penetrate to target the cells has increased [32]. Penetrability is expected to decrease as the size of the probe increases, but these factors affect even the distribution of small (≤0.5 kDa) molecules. Although high uptakes of Affibody molecules have been obtained in tumors due to a favorable balance between their high affinity and smaller size compared to antibodies and fragments, [33] hypoxia and collapse of vessels due to increasing pressure would clearly contribute to a disparity between the total receptor levels and those measured by the in vivo imaging tracers.

In the longitudinal A431 study, the uptake of the non-targeting protein did not vary significantly, probably because the tumors were still small and all inoculations were from the same cell culture to minimize variations in initial angiogenic status [34]. On the group level, the specific uptake increased with time from inoculation (Fig. 6d). However, on the individual level, there was a tendency for uptake to level off as sizes increased in several of the tumors (Fig. 6f)). This has previously also been observed with EGFR-targeting 15-kDa nanobodies in larger A431 xenografts and was suggested to be due to necrosis and/or decreasing penetration of the probe [35]. However, the A431 tumors studied here were purposefully kept small, and necrotic areas with heterogeneous tracer uptake were not discernible. According to the PLA analyses, total EGFR levels tended to increase with the size of tumors (Fig. 7b) but did not positively correlate to increases in specific tracer uptake (Fig. 7c). Previous studies have also reported that ex vivo assays of EGFR expressions did not correlate with the in vivo uptake of other targeting ligands (e.g., [36–38]). There are a number of reasons why discrepancies could be expected [4]. For example, ex vivo assays are optimized so maximum binding of the labeled probes can be obtained in the excised sections and are not performed under the same conditions that affect probe accessibility in vivo. Also, they generally only sample a small part of the tumor and are therefore not necessarily representative of the whole tumor. On the other hand, in vivo tracer measurements (like PET) of whole tumor volumes are affected by tracer ability to overcome in vivo pharmacological barriers in order to effectively bind. These techniques also have much lower resolution, so all the details observable with techniques like IHC and PLA are not discernible. The PLA analyses performed here revealed that the EGFRs in the tumors were localized on the membrane as well as in the cytoplasm. Z_EGFR:2377_ binds to membranous EGFR and is barely internalized in A431 during at least 5 hrs [16], although the degree of internalization may vary depending on the tumor and time [15]. Since we only monitored the ^11^C-labeled Z_EGFR:2377_-based probe during a 1-hr scan, it was probably primarily binding to available membranous receptors. Therefore, no correlation to the total (membranous and cytoplasmic) expression levels revealed with IHC and PLA techniques should be expected.

Receptors are not static systems. For example, fluxes in the levels of endogenous ligands influence the receptors that are available for binding by radioligands as well as by receptor-targeting pharmaceuticals. Ligand/receptor interactions can lead to changes in the localization of the receptors. Influences of the endogenous ligand on levels of dopamine receptors have been actively studied with PET (e.g., [39]), and this is also an area of active research on other neuroreceptor systems. The dynamics of receptor availability are likewise very important in many aspects of cancer research. EGFR dimerization, internalization, receptor down-regulation, and degradation are all major determinants of the nature and duration of the receptor signaling [40]. Differences in the membranous vs. cytoplasmic locations of EGFRs have been suggested to be associated with tumorigenesis and a prognostic factor for overall survival (e.g., [41, 42]). The availability of appropriate in vivo imaging tools could be very valuable for probing these differences in order to better understand factors affecting the dynamics of the receptor cyclisations, similar to studies performed in neuroreceptor PET. Furthermore, many anti-EGFR drugs target the membranous receptor. Therefore, imaging probes that monitor the availability of these targets can be important for making prognoses about who would benefit from these therapeutic strategies. It has been suggested that EGFR status in vivo might be studied in serial biopsies of normal and tumor tissues [4]. We suggest instead that this serial dual Affibody-based tracer imaging technique, possibly in tandem with tracers such as ^11^C-labeled erlotinib [43] that target the EGFR tyrosine kinase domain (and therefore estimate the receptors in both the membrane and cytoplasm), could provide non-invasive methods for studying changes in numbers and locations of receptors during tumor growth and EGFR cycling.

## Conclusions

This study demonstrated the feasibility of using a dual PET tracer strategy using EGFR-targeting [methyl-^11^C]-Z_EGFR:2377_-ST-CH_3_ followed by the size-matched control [methyl-^11^C]-Z_Taq:3638_-ST-CH_3_ to study EGFR levels and examine underlying factors contributing to the imaging read-out. Though the specific uptake did differ for tumors with different EGFR expression levels, the uptakes were also affected to different degrees by factors in the tumor architecture limiting their access and non-specific uptake mechanisms, as well as whether the EGFRs to be quantified are localized extra- or intracellularly. This tracer pair appears promising for further applications as investigational imaging biomarkers in PET studies of fluctuations in accessible membrane EGFRs.

## Methods

### DNA constructions and expression of Sel-tagged Affibody molecules

The EGFR-binding Affibody molecule Z_EGFR:2377_ [15, 16] and the irrelevant *Taq* polymerase-binding Affibody molecule Z_Taq:3638_ [18] were fused with a C-terminal ST as previously described [19, 44]. Gene segments encoding a hexahistidyl tag (H_6_), GFP, and a cleavage site for TEV-protease were introduced upstream of the Affibody molecules using polymerase chain reaction (PCR), to encode the constructs H_6_-GFP-TEV-Z_EGFR:2377_-ST and H_6_-GFP-TEV-Z_Taq:3638_-ST. Sequence-confirmed plasmids were transformed to BL21(DE3) cells (Novagen) already harboring the pSUABC plasmid [45]. The two fusion proteins were expressed in shake flasks (LB medium supplemented with 50 μg/mL kanamycin and 34 μg/mL chloramphenicol. Gene expression was induced by adding isopropyl β-D-1-thiogalactopyranoside to a final concentration of 0.5 mM. l-Cysteine and selenite were added to the cultures to a final concentration of 1 μM and 5 nM, respectively, and cultivations were continued overnight at 25 °C. Cells were harvested by centrifugation at 5,000 rpm and disrupted by sonication followed by centrifugation 16,000 rpm, 20 min, 4 °C. The two fusion proteins were purified by IMAC on a HisTrap FF column (GE Healthcare) and eluted with 50 % tris(hydroxymethyl)aminomethane hydrochloride (Tris)-HCl buffer, 50 mM, pH 8.0, containing 500 mM imidazole. Buffer was exchanged to TEV cleavage buffer (50 mM Tris-HCl pH 8, 0.5 M ethylenediaminetetraacetic acid (EDTA), 1 mM dithiothreitol (DTT)) by dialysis prior to digestion by TEV protease overnight at 4 °C using 50-times excess of a His-tagged TEV protease. Cleavage mixtures were applied on IMAC columns, and Affibody molecules were recovered in the flow-through. Protein purity was assessed by SDS-PAGE analysis. Z_EGFR:2377_-ST and Z_Taq:3638_-ST were further purified by reversed phase chromatography using a Resource RPC 1-ml column (GE Healthcare), using the following buffer system: buffer A, 0.1 % trifluoroacetic acid (TFA) in water, and buffer B, 0.1 % TFA in acetonitrile. The protein was eluted with a gradient of 5–50 % buffer B over 20 min.

### Fluorescence labeling to produce Z_EGFR:2377_-ST- and Z_Taq:3638_-ST-[DyLight488]

Z_EGFR:2377_-ST and Z_Taq:3638_-ST were labeled specifically at the Sec residue using maleimide-activated DyLight 488 (Pierce). Briefly, Sel-tagged Affibody (30 μM) was reduced with DTT (1 mM) at room temperature for 30 min and then incubated in the dark with DyLight 488 (2.5 mM, dimethylsulfoxide (DMSO)) for 30 min. DTT (5 mM) was added to quench the reaction, and the Affibody-ST-[DyLight488] was purified and desalted to physiologically buffered saline (PBS) through a NAP-5 column (GE Healthcare). The concentration of the Z_EGFR:2377_-ST-[DyLight488] or Z_Taq:3638_-ST-[DyLight488] was determined spectrophotometrically using an extinction coefficient of 21,050 M^−1^cm^−1^ at 280 nm by a Nanodrop (Thermo). The labeled proteins were stored in the refrigerator without direct exposure to light.

### ^11^C-Labeling to produce [methyl-^11^C]- Z_EGFR:2377_-ST-CH_3_ and -Z_Taq:3638_-ST-CH_3_

The Sel-tagged Affibody molecules were ^11^C-labeled specifically at the Sec residue, by the method used previously [19, 20, 29, 46]. Briefly, the Sel-tagged Affibody molecule was reduced with DTT (1 mM) at 35–37 °C for ≥20 min. An aliquot (10–25 μl) of [^11^C]CH_3_ (prepared as in [47] from cyclotron-produced [^11^C]CO_2_ (PETtrace, GE Healthcare)) and trapped in DMSO (0.2 ml)) was added. After 20 min at 35–37 °C, the reaction was quenched with DTT (5 mM) and the [methyl-^11^C]-Affibody-ST-CH_3_ was desalted (NAP-5, PBS). Radiochemical purity was analyzed by radio-HPLC (Superdex Peptide 10/300 GL (GE Healthcare) and eluted with PBS, using ultraviolet- (210 nm) and radio-detectors in series).

### Cell lines and animals

All cell lines were purchased from American Type Culture Collection and cultured at 37 °C, supplemented with 5 % CO_2_ in a humidified environment. A431 and FaDucells were cultured in Dulbecco’s Modified Eagle’s Medium (DMEM) (4500 mg/L d-glucose containing l-glutamine) whereas MDA-MB-453 was cultured in DMEM (1000 mg/L d-glucose containing l-glutamine). All media were additionally supplemented with 1 mM sodium pyruvate, 100 units penicillin/mL, 100 μg streptomycin/mL, and 10 % foetal bovine serum. Media for FaDu cells were further supplemented with 0.1 mM non-essential amino acids and 2 mM HEPES. EGFR expression levels were confirmed using western blotting (Fig. 1c). The integrity of the cell lines was verified with short tandem repeat (STR) profiling cell authentication analysis (LGC Standards).

Balb/c, SCID, and Balb/c (nu/nu) mice were purchased from Charles River. The animals were housed under standard conditions according to local regulations, with access to food and water ad libitum in the Department of Comparative Medicine at Karolinska University Hospital, Solna.

### Cell-binding assay using fluorescence-activated cell sorting (FACS) analysis

Cells were trypsinized, washed with PBS containing 1 % albumin, divided into 200 μl aliquots containing 300,000 cells, which were then incubated 1 h with 0.1 μM of either Z_EGFR:2377_-ST-[DyLight488] or Z_Taq:3628_-ST-[DyLight488]. The labeled cells were subsequently separated from non-bound tracer by centrifugation. PBS buffer was used as a negative control. For blocking experiments, cells were pre-incubated with 1 μM non-labelled Z_EGFR:2377_-ST for 5 min before adding 0.1 μM Z_EGFR:2377_-ST-[DyLight488]. After shaking for 1 h at room temperature, the cells were washed with PBS containing 1 % albumin, suspended in PBS and then analysed with a FACSort Calibur flow cytometer (Becton Dickinson).

### Ex vivo fluorescent imaging of liver, kidney, and xenograft sections

A431 cells (10^7^ in 0.15 ml PBS) were inoculated subcutaneously (s.c.) into the right flank of four mice (Balb/c, nu/nu, female, 18–20 g, 10 weeks old). After 3 weeks, the mice were injected intravenously (i.v.), in the tail vein with either PBS, 60 μM Dylight 488 dye, 60 μM Z_EGFR:2377_-ST-[DyLight488], or 60 μM Z_Taq:3638_-ST-[DyLight488] (0.2 ml). Mice were sacrificed 1-h postinjection (p.i.), and tissues were excised, snap frozen (−78 °C), and sectioned (25 μm) using a freezing microtome. The fluorescence was documented using an Axio Observer.Z1 fluorescence microscope (Zeiss).

### Western blotting

Western blotting was performed using fresh cell lysates or snap frozen tumor tissue excised from the animal. Briefly, cells or tumor was first homogenized with lysis buffer (50 mM Tris-HCl, pH 7.5, 5 mM EDTA, 150 mM NaCl, 1 % Triton X-100) in the presence of protease inhibitor, and then three cycles of freeze-thawing were applied on the mixture. The insoluble components including cell or tissue debris were removed by centrifugation. The supernatant corresponding to 20 μg of total protein was analyzed by western blotting (Life technology) using an antibody against human EGFR (Santa Cruz, sc-03, 1:200). The reaction product was detected by enhanced chemiluminescence (Perkin Elmer).

### Immunohistochemistry

Tissues were resected, fixed in 2 % buffered formaldehyde, dehydrated, embedded in paraffin, and sectioned. The sections were then deparaffinized with xylene, rehydrated, and microwaved and then incubated overnight with the monoclonal primary antibodies diluted in 1 % (wt/vol) BSA and visualized by standard avidin–biotin–peroxidase complex technique (Vector Laboratories, Burlingame, CA, USA). Counterstaining was performed with Mayer’s hematoxylin. The antibody against EGFR (Sigma, Sweden) was used at a dilution 1:800, and the antibody against CAIX (BioScience, Slovakia) was used at 1:500.

### Phosphoimaging

After the mice were sacrificed, tumors were removed and immediately frozen in dry ice and sectioned using a cryomicrotome (CM 3050S, Leica Microsystems, Wetzlar, Germany) with a thickness of 25 μm. The sections were exposed on phosphor imaging plates for at least 1 hour. Scanning was performed using a Typhoon FLA 7000 (GE Healthcare).

### Proximity ligation assays

The PLA protocol [48–50] is the standard procedure by which the in situ PLA assay is performed at the proteomics facility, Uppsala, Sweden *(**http://www.scilifelab.se/facilities/pla-proteomics**)* and was followed according to manufacturers’ instructions (Olink Bioscience, Uppsala, Sweden). Briefly, formalin-fixed paraffin-embedded tumor tissue sections (4 μm) were deparaffinised, and the antigen retrieval procedure was performed according to protocol using 1xTarget retrieval solution citrate pH 6 (Dako art, S2369). After blocking, tumor sections were incubated over night (4 °C) with primary antibodies EGFR (D38B1) XP® Rabbit mAb #4267 (Cell Signalling) and mouse monoclonal EGFR (E3138, Sigma-Aldrich), diluted 1:800 with Duolink diluent solution (Sigma-Aldrich). After incubation, the slides were washed in TBS-T, 2 × 5min.

PLA secondary probes anti-rabbit MINUS and anti-mouse PLUS (Duolink kit, Sigma), containing the secondary antibodies conjugated with oligonucleotides, were diluted 1:5 in Duolink antibody diluent. The secondary probe mixture (40 μl) was added to each sample, and the slides were incubated in a humidity chamber for 1 h at 37 °C. The slides were washed in TBS-T, 2 × 5min. Following the protocol, the ligation solution was added to each sample, and the slides were incubated for 30 min at 37 °C. After incubation, the ligation solution was tapped off, the slides were washed in TBS-T, and 40 μl of amplification solution was added to each sample followed by incubation for 100 min at 37 °C. Hoechst 33342 dye nuclear staining (Life Technologies, H1399) was added and incubated for 1 h at room temperature. After washing in MQ water over night, 15 μl of SlowFade Gold Antifade Reagent (Invirogen, S36936) was added to each sample and covered with a cover slip. The positive PLA signals were visualized as distinct red dots in epifluorescence microscope Axioplan 2 (Zeiss). Stacked TIFF images were acquired using Axiovision 4.8. Data analysis for in situ PLA images was performed using the freeware Cell Profiler, developed by the Broad Institute *(**http://www.cellprofiler.org**)*. The data is given in PLA signals/cell. For PLA signal quantification, three images were taken of each section at ×20 magnification, one each of positive (with high density of fluorescence signals), one of the border between positive and negative tissues, and one of negative tissue (with low concentration of fluorescence signals). The number of PLA signals per image per single cell was estimated as a ratio between total fluorescence signal and total number of cells. At less than two PLA signals per single cell, tissues were counted as a negative.

### PET data collection and processing

PET imaging was performed using a microPET Focus 120 scanner (CTI Concorde Microsystems, Knoxville, TN, USA). List mode data, collected continuously over 60 min from the tracer injection, were reconstructed using Ordered Subset Estimation Maximum in 2 dimensions (OSEM2D) to increase the spatial resolution, with a picture size of 256 × 256 pixels, four iterations, and 16 subsets. Data, normalized and corrected for randoms, dead time, and radioactivity decay, were processed using MicroPET Manager and evaluated using the Inveon Research Workplace (IRW) software (Siemens Medical Systems, Malvern, PA, USA). Venous and arterial ROIs were delineated on the vena cava and left ventricle of the heart, respectively, in the first frames of the scan. Muscle ROIs were drawn on the right hamstring muscle (images summed 0–60 min). ROIs of same shape and size were drawn on the renal cortex and liver, on images summed over periods of maximum uptake after the initial distribution, 10–20 min p.i. Tumor ROIs were delineated on images summed between 30 and 60 min and thresholded ≈75 %, based on palpation and subsequent post-mortem measurements. The IRW software calculated the SUV_mean_ and SUV_max_ and their standard deviations, along with the volumes of the ROIs (here called the “image-derived volumes,” as opposed to those based on measurements by palpations).

### PET imaging of [methyl-^11^C]-Z_EGFR:2377_-ST-CH_3_ and -Z_Taq:3638_-ST-CH_3_

All experiments involving animals were conducted in accordance with the regulations of the Karolinska Institutet and approved by the local laboratory animal ethics committee (N325/09, N85/11, N416/12). Mice were anesthetized (1.5 % isoflurane (Virbac) blended with air (7:3) in a vaporizer (E-Z systems) delivered through a Microflex non-rebreather mask (Euthanex Corp)) and intravenously (i.v.) injected (tail vein) with [methyl-^11^C]-Affibody-ST-CH_3_. Radioactivity in regions of interest (ROIs) was calculated as mean and maximum standardized uptake values (SUV_mean_ and SUV_max_) normalized to body weight [51]. The uptake of the non-targeting ligand was subtracted from that of the targeting ligand as a measure of specific uptake in some of the analyses.

For the biodistribution studies, Balb/c mice (female, 18–20 g) were i.v. injected with [methyl-^11^C]-Z_EGFR:2377_-ST-CH_3_ or [methyl-^11^C]-Z_Taq:3638_-ST-CH_3_ (volume ≤0.2 ml). Following previous studies on the effects of the administering masses in the interval of 0.1–150 μg [16], the masses of the Z_EGFR:2377_-based ligand were adjusted or spiked with unlabeled Z_EGFR:2377_ to lower the specific activity (here a total mass of 27 or 49 μg, (*n* = 2 each)). The latter Z_Taq:3638_-based ligand (*n* = 4) was not spiked. The necessity for adjusting the targeting protein mass to reduce non-tumor uptake so tumors could be visualized was confirmed in two FaDu-bearing mice (Additional file 1: Figure S1). Thereafter, for studies in the tumor-bearing mice, the protein in the radiolabeling vial was adjusted so the mass of targeting tracer injected would be 50–100 μg, in accordance with the recommended dose interval previously established in [16].

In the first pilot studies, mice with single and double inoculations of FaDu and A431 cells were studied. Imaging was performed with both the targeting and non-targeting tracer, except in the doubly inoculated FaDu-bearing mice. In those mice, only investigations with [methyl-^11^C]-Z_EGFR:2377_-ST-CH_3_ were performed to see if the uptakes would follow the growth of the tumors. When this was found to not to be the case, only the A431 tumors were chosen for the longitudinal study.

For the study of tumors with low/intermediate EGFR expression, FaDu cells were injected subcutaneously (s.c.) in SCID mice (male, 18–29 g, single inoculations, *n* = 4 mice, with 1 × 10^6^ cells; double inoculations, *n* = 4 mice, with 1 × 10^6^ and 0.5 × 10^6^ cells on the left and right shoulders, respectively). FaDu xenografts grow very rapidly once they have established, and for this reason, the sizes of the xenografts were varied by performing the double inoculations on the same day, but with different numbers of cells. After 12–13 days, PET imaging was performed with [methyl-^11^C]-Z_EGFR:2377_-ST-CH_3_ and, for mice with single xenografts, ≥3.5 h later with [methyl-^11^C]-Z_Taq:3638_-ST-CH_3_. The time interval between the two investigations was chosen in order to allow sufficient time (≥8 half-lives) for the decay of the radioactivity from the previous investigation. Tumors excised after sacrifice were prepared for analyses by PI and IHC.

A431 cells (high EGFR expression) were injected s.c. in Balb/c, nu/nu mice (female, 16–24 g, single inoculation in four mice, 10^7^ cells; two inoculations in five mice, 3 days apart to allow the mice to recover between inoculations, first on the left and then on the right shoulder, 10^7^ cells each time). A431 xenografts grow more slowly than FaDu, and therefore, tumors at different stages of development could be observed by spreading out the interval between the first and second inoculations. PET imaging was performed with [methyl-^11^C]-Z_EGFR:2377_-ST-CH_3_ and ≥3.5 h later [methyl-^11^C]-Z_Taq:3638_-ST-CH_3_. Scanning started from when the xenografts were palpable (≥8 days) and thereafter, in the longitudinal study, 2–11 days between the scanning sessions, depending on xenograft development. Restricted by logistics and the scans allowed by the ethical permission, two to five scans/animal were performed. Details are given in the Additional file 2: Table S1. Excised tumors were subsequently prepared for analyses by PI, IHC, and/or PLA.

### Statistics

Imaging results are presented as values ± SD, if not otherwise specified. Deviations of the measured values from fitted lines were estimated using the Pearson’s correlation coefficient, *r*^2^, ranging from 0–1, where 1 is a total positive and 0 is no correlation between the variables *X* and *Y*.

## Abbreviations

CAIX, carbonic anhydrase IX; Da, Dalton; DMEM, Dulbecco’s Modified Eagle’s Medium; DMSO, dimethylsulfoxide; DTT, dithiothreitol; EDTA, ethylenediaminetetraacetic; EGFR, epidermal growth factor receptor; EPR, enhanced permeability and retention; FACS, fluorescence-activated cell sorting; GFP, green fluorescent protein; HPLC, high-performance liquid tomography; i.v., intravenous; IHC, immunohistochemistry; PBS, physiologically buffered saline; PCR, polymerase chain reaction; PET, positron emission tomography; PI, phosphoimaging; PLA, proximity ligand assay; PVE, partial volume effect; s.c., subcutaneous; SCID, severe combined immunodeficiency; SD, standard deviation; Sel, selenocysteine; SPECT, single photon emission computed tomography; ST, sel-tag; SUV, standardized uptake value; TAC, time activity curve; TEV, tobacco etch virus; TFA, trifluoroacetic acid

## Additional files

Additional file 1: Figure S1. PET image, summed 40–60 min, of the uptake of targeting [methyl-11C]-ZEGFR:2377-ST-CH3 in one SCID mouse (prone) bearing one s.c. FaDu tumor (1 × 106 cells, 13 days). Only 16-μg protein was injected and nearly all radioactivity localized very quickly in the liver giving SUV mean >2.5 times larger than for animals receiving spiked tracer injections. The tumor (*white arrow*) was barely discernible with a faint vascular signal. Even radioactivity distributing to the kidneys and urinary bladder during the 60 min was markedly reduced. Thereafter, the amount of protein administered was adjusted to 50–100 mg to partially block the hepatic uptake and free the ligand for tumor targeting, as discussed in the text. (PPTX 422 kb) Additional file 2: Table S2. Imaging information and growth details of the A431 tumors included in the longitudinal study. (DOCX 17 kb)
