# Supplementary material for: Preclinical PET imaging of EGFR levels: pairing a targeting with a non-targeting Sel-tagged Affibody-based tracer to estimate the specific uptake
Source: EJNMMI Res. 2016 Jul 7;6:58. doi: 10.1186/s13550-016-0213-8 (PMC4936982; doi:10.1186/s13550-016-0213-8)
Supplement: Additional file 1: Figure S1. — PET image, summed 40–60 min, of the uptake of targeting [methyl-11C]-ZEGFR:2377-ST-CH3 in one SCID mouse (prone) bearing one s.c. FaDu tumor (1 × 10^6^ cells, 13 days). Only 16-μg protein was injected and nearly all radioactivity localized very quickly in the liver giving SUV mean >2.5 times larger than for animals receiving spiked tracer injections. The tumor (white arrow) was barely discernible with a faint vascular signal. Even radioactivity distributing to the kidneys and urinary bladder during the 60 min was markedly reduced. Thereafter, the amount of protein administered was adjusted to 50–100 mg to partially block the hepatic uptake and free the ligand for tumor targeting, as discussed in the text. (PPTX 422 kb) [file 13550_2016_213_MOESM1_ESM.pptx]

## Slide 1
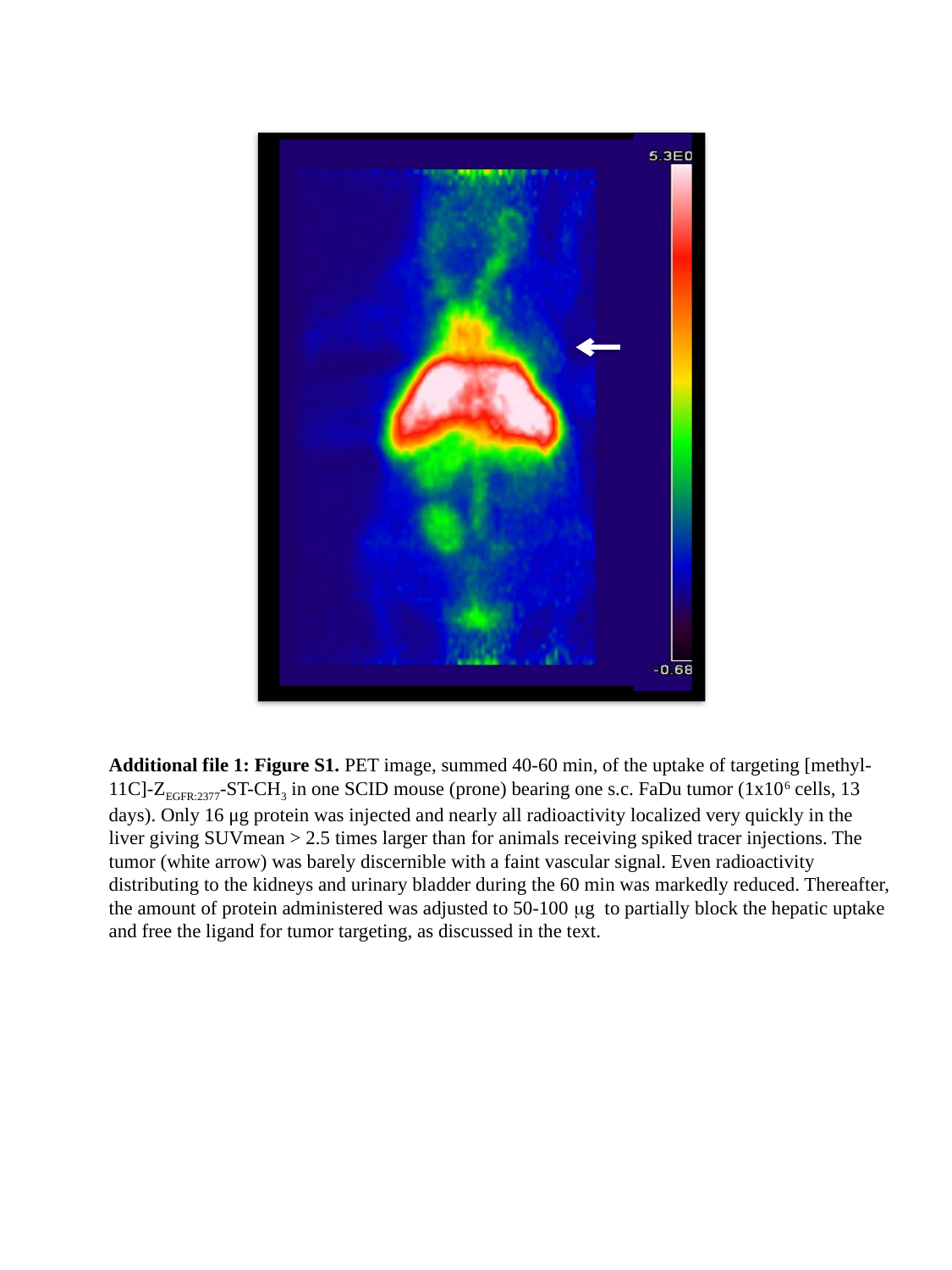

Additional file 1: Figure S1. PET image, summed 40-60 min, of the uptake of targeting [methyl-11C]-ZEGFR:2377-ST-CH3 in one SCID mouse (prone) bearing one s.c. FaDu tumor (1x10^6^ cells, 13 days). Only 16 μg protein was injected and nearly all radioactivity localized very quickly in the liver giving SUVmean > 2.5 times larger than for animals receiving spiked tracer injections. The tumor (white arrow) was barely discernible with a faint vascular signal. Even radioactivity distributing to the kidneys and urinary bladder during the 60 min was markedly reduced. Thereafter, the amount of protein administered was adjusted to 50-100 mg to partially block the hepatic uptake and free the ligand for tumor targeting, as discussed in the text.
