# Supplementary material for: Preclinical PET imaging of EGFR levels: pairing a targeting with a non-targeting Sel-tagged Affibody-based tracer to estimate the specific uptake
Source: EJNMMI Res. 2016 Jul 7;6:58. doi: 10.1186/s13550-016-0213-8 (PMC4936982; doi:10.1186/s13550-016-0213-8)
Supplement: Additional file 2: Table S2. — Imaging information and growth details of the A431 tumors included in the longitudinal study. (DOCX 17 kb) [file 13550_2016_213_MOESM2_ESM.docx]

**Additional file 2: Table S1.** Imaging information and growth details of the A431 tumors included in the longitudinal study

| **Mouse#-tumor position** | **Date**  **Days from innoculation** | **Tracer(s) imaged** | **Palpated dimensions**  **(mm)** | **Date**  **Days from innoculation** | **Tracers(s) imaged** | **Palpated dimensions**  **(mm)** | **Date**  **Days from innoculation** | **Tracer(s) imaged** | **Palpated dimensions**  **(mm)** |
| --- | --- | --- | --- | --- | --- | --- | --- | --- | --- |
| #1-left ^a^ | 11 days | Z2377 + ZTaq | 2 x 2 ^b^ | 22 days | Z2377 + ZTaq | 3 x 3 | 24 days | Iv injection not possible, mouse sacrificed without imaging | |
| #1-right ^a^ | 8 days |  | 3 x 4 | 19 days |  | 3.5 x 4 | 21 days |  |  |
|  |  |  |  |  |  |  |  |  |  |
| #2-left ^a^ | 11 days | Z2377 + ZTaq | 1 x 1.5 ^b^ | 22 days | Z2377 + ZTaq | 3 x 3.5 | 24 days | Z2377 ^c^ | 4.5 x 5.5 |
| #2-right ^a^ | 8 days |  | 3.5 x 4.5 | 8 days |  | 3 x 3.5 | 21 days |  | 3 x 4 |
|  |  |  |  |  |  |  |  |  |  |
| #3-left | 18 days | Z2377 + ZTaq | 3 x 3 | 21 days | Z2377 + ZTaq | 4 x 5 | 23 days | Z2377 ^c^ | 5 x 5 |
| #3-right | 15 days |  | 3 x 3 | 18 days |  | 4.5 x 4.5 | 20 days |  | 5 x 5 |
|  |  |  |  |  |  |  |  |  |  |
| #4-left | 18 days | Z2377 + ZTaq | 3.5 x 4.5 | 21 days | Z2377 + ZTaq | 4 x 4.5 | 23 days | Z2377 ^c^ | 5 x 5.5 |
| #4-right | 15 days |  | 3.5 x 3.5 | 18 days |  | 4 x 4.5 | 20 days |  | 4.5 x 5 |
|  |  |  |  |  |  |  |  |  |  |
| #5-left | 28 days | Z2377 + ZTaq | 5 x 7.5 | Mouse sacrificed without further imaging due to total tumor burden. | | | | | |
| #5-right | 25 days |  | 3.5 x 5 |  |  |  |  |  |  |

^a^ Mouse #1 and #2 were excluded from the PLA performed post-sacrifice, due to problems with the i.v. injections on the final imaging session that did not allow imaging of #1 and might possibly have affected the second. Tumors from all other mice were included in the PLA analyses.

^b^ According to measurements by palpation, sizes in two tumors on first imaging day were close to the resolution of the scanner and partial volume effects may have affected the calculated radioactivity concentrations.

^c^ Since ethical permission for this study only allowed five PET imaging sessions per mouse, the last imaging session was only performed with the EGFR-targeting ligand. Since levels of uptake of the non-targeting ligand did not vary greatly in all the A431 tumors imaged in this longitudinal study, for this session compared an average value for the non-targeting ligand was used in the estimations of specific uptake.
